# A Dynamic Ensemble Framework for Mining Textual Streams with Class Imbalance

**DOI:** 10.1155/2014/497354

**Published:** 2014-04-10

**Authors:** Ge Song, Yunming Ye

**Affiliations:** Shenzhen Key Laboratory of Internet Information Collaboration, Shenzhen Graduate School, Harbin Institute of Technology, Shenzhen 518055, China

## Abstract

Textual stream classification has become a realistic and challenging issue since large-scale, high-dimensional, and non-stationary streams with class imbalance have been widely used in various real-life applications. According to the characters of textual streams, it is technically difficult to deal with the classification of textual stream, especially in imbalanced environment. In this paper, we propose a new ensemble framework, clustering forest, for learning from the textual imbalanced stream with concept drift (CFIM). The CFIM is based on ensemble learning by integrating a set of clustering trees (CTs). An adaptive selection method, which flexibly chooses the useful CTs by the property of the stream, is presented in CFIM. In particular, to deal with the problem of class imbalance, we collect and reuse both rare-class instances and misclassified instances from the historical chunks. Compared to most existing approaches, it is worth pointing out that our approach assumes that both majority class and rareclass may suffer from concept drift. Thus the distribution of resampled instances is similar to the current concept. The effectiveness of CFIM is examined in five real-world textual streams under an imbalanced nonstationary environment. Experimental results demonstrate that CFIM achieves better performance than four state-of-the-art ensemble models.

## 1. Introduction

Delivering emails and forums, providing chatting rooms, and publishing blogs are making textual streams dynamic and large scale. It is a big challenge for the textual stream classification because of many interesting characteristics of a textual stream. First, a textual stream with large-scale data instances is of high-dimensional and imbalanced distribution. High-dimensional feature distribution is a persistent problem in general data classification contexts. In addition, skewed class distributions can be seen in many data stream applications, such as credit card transactions and intrusion detection. In these cases, the instances in some classes (rare classes), which gain more attention and are of more importance in most of the real-world application, are much more difficult to be gathered than the other classes. In traditional existing classification models, imbalanced distribution may cause performance degradation since building these models mainly relies on instances in majority classes, ignoring the importance of the rare class. Therefore, most existing data stream classification methods fail to tackle the textual stream due to both its high-dimensional feature space [1] and imbalanced class distribution. Second, concept drift may occur in the period of time. This property requires the current classifier to fit up-to-date concepts. For instance, the customers' buying preferences and patterns may change with time. This drives us to develop an adaptive model to meet the current buying preferences and patterns of customers.

Ensemble methods, which are regarded as promising methods to deal with textual streams, aim at integrating several individual submodels to form a final prediction [2]. It is easy to obtain training set for each submodel since a stream can naturally be divided into several individual chunks. In this paper, we propose a new ensemble model, clustering forest, to classify textual stream with imbalanced and drifting stream (CFIM). CFIM aims at integrating a number of clustering trees (CTs) by an efficient and adaptive method. It is worth noting that CT, a clustering-based classification algorithm [3], is suitable for large high-dimensional sparse textual data [3]. Besides the current chunk for training the submodels, we collect another two new subsets, rare-class subset and misclassified subset, to tackle the imbalanced problem. These two new subsets are aggregated from the historical chunks with the same feature distribution. It is worth pointing out that compared with other existing imbalanced stream classification approaches, which assume that the distribution of rare class is not changed [4], the assumption of our model is that all the instances in the stream may suffer from concept drift and change their feature distribution. A suitable number of CTs are combined automatically to accomplish the optimal prediction result. During the ensemble process, we propose an adaptive selection method. A dynamic threshold is used to measure whether the CT fits a new concept. The threshold is defined according to the accuracy weight of a CT rather than using the random prediction accuracy by most of the existing models [5, 6]. The performance of CFIM is experimented on several textual stream datasets on Massive Online Analysis (MOA) platform [7]. Compared with other four state-of-art ensemble approaches, experimental results show that CFIM delivers the promising performance regarding the average accuracy, the plotting accuracy, and the kappa statistic.

The previous works related to our study are reviewed in Section 2; we then present the overall framework of CFIM in Section 3. In Section 4, we propose the sampling method to form training set. The adaptive selection method and voting method are proposed in Section 5. Experimental results are analyzed in Section 6. In Section 7, we summarize the whole paper and give suggestions on future work.

## 2. Related Work

### 2.1. Textual Stream with Class Imbalance

In various real-life applications, textual stream is characterized by having the high-dimensional feature space, a large number of instances with imbalanced distribution and the concept drift. Many researches of ensemble learning focus on textual stream classification with concept drift [6, 8–11]. For example, the Conceptual Clustering and Prediction (CCP) [11] framework has been proposed to classify the textual stream with recurring concept. Incremental subclassifiers in CCP were constructed and updated when new training instances arrived. A new subclassifier was built when a new concept was detected. Thus one concept corresponded to only one incremental subclassifier. In this sense, it was not an ensemble approach in the period of one concept. Moreover, a semisupervised approach, PU Learning by Extracting Likely Positive and Negative Microclusters (LELC) [9], aimed at dealing with positive and unlabeled textual stream problems. The extension research of LELC, Voted LELC [12], was then presented. This method allocated a voting weight to each representative instance. The voting weight represented the belongingness of an instance towards its corresponding class. Then Voted-LELC combined all SVM-based subclassifiers (built by voted representative instances) into global prediction result using the AWE ensemble model. To overcome some drawbacks of AWE, this method applied active learning to classify unknown textual instances. Similar approach to one-class classification of textual streams was proposed by Zhang et al. [10].

Besides the concept drift, many researches aimed at overcoming another issue, class imbalance. A framework [13, 14] based on collecting rare-class examples has been proposed. All the instances in the rare class should be gathered into the training chunk, while the instances belonging to the majority class were sampled into the training set according to a distribution radio. This approach implicitly assumed that the distribution of rare class is not drifting over time [4]. Likewise, Chen and He [15] have proposed SERA algorithm, which selected the “best” rare-class instances according to the Mahalanobis distance, instead of using all old instances in some algorithms, such as [13, 14]. However, the algorithm may not be able to track drift in rare-class instances. Another research based on [13, 14] has been proposed by Lichtenwalter and Chawla [16]. In their paper, besides collecting rare-class instances, they also collected the misclassified majority class instances. In addition, Learn++.NIE (for learning in nonstationary and imbalanced environments) [4, 17, 18] has been proposed to deal with class imbalance. Compared with the original Learn++.NSE algorithm, a step using the bagging algorithm was added to handle the imbalance problem.

### 2.2. Features of Our Approach

Our new ensemble approach, CFIM is proposed to handle textual streams with class imbalance and concept drift. CFIM is different with other traditional ensemble models from the following aspects.

- According to the adaptive selection method, CFIM changes the number of selected submodels along with the variation of the up-to-date concept. It is more flexible to select suitable submodels in comparison to many existing methods with the fixed number of selecting the submodels.
- We accumulate rare-class instances and misclassified instances from the historical chunks with the same feature distribution to better define the boundary between the classes. It is worth mentioning that our CFIM assumes that both majority class instances and rare-class instances may suffer from concept drift. Therefore, the instances in rare-class subset and misclassified subset should be sampled from the historical chunks, whose distributions are as similar as the latest chunk. Moreover, based on the sample method in our CFIM, the majority class instances should be sampled at a proper radio to form balanced training set.

## 3. Clustering Forest for Imbalanced and Drifting Stream (CFIM)

**Figure 1:**
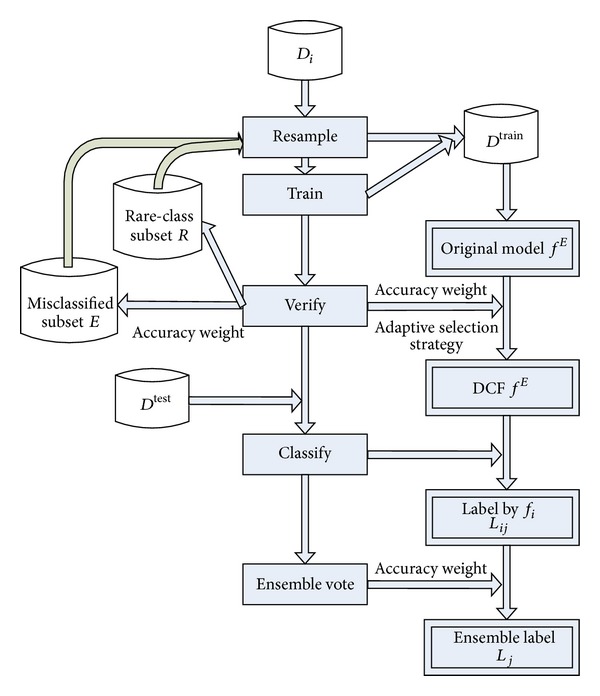
Framework of clustering forest (CFIM).

In this paper, we propose a new ensemble model, CFIM, for imbalanced and drifting textual stream. The framework of our model is shown in Figure 1. CFIM includes four steps:

- Resample step, which is to form the training chunk by combining the new chunk with misclassified subset *E* and rare-class subset *R* according to sample method.
- Training step, which is to train the submodels, CTs.
- Verifying step, which is to adaptively select the CTs and form the misclassified subset *E* and rare-class subset *R*.
- Testing step, which is to predict the labels of the incoming testing instances.

The verifying step consists of three substeps.

1. Compute the accuracy weight of each CT.
2. Adaptively select the CTs to integrate the optimal CF.
3. Form the misclassified subset *E* and rare-class subset *R* by accuracy weight.

The testing step consists of two substeps.

1. Predict the labels of incoming testing instances by each CT in CFIM.
2. Integrate the CTs and obtain the global prediction according to accuracy weight.

We would like to clarify the framework of CFIM as follows (the details of CFIM are shown in Algorithm 1).

- Form the training set. The training set consists of new training chunk *D*_*t*_, the misclassified subset *E*_*t*_, and the rare-class subset *R*_*t*_. We would like to define the radio *p* ^*R*^ which is the size of rare-class subset as a proportion of the training chunk *D*_*t*_^train^, *p* ^*R*^ = |*R*|/|*D*_*t*_^train^|. The threshold *p*_*θ*_^*R*^ is introduced to control the number of majority class instances. At the *t*th time stamp, if *p*_*t*_^*R*^ < *p*_*θ*_^*R*^, that means the number of rare class is too small to influence the construction of submodel. So all the instances in rare class should be added in the training set at this time stamp. Meanwhile, we should undersample the incoming chunk *D*_*t*_ and select several ((1 − *p*_*θ*_^*R*^)|*D*_*t*_|) majority class instances to balance the number of rare class and majority class.
- Build CTs to integrate the original clustering forest (CF). Since the submodels have time limited effectiveness, *M*_max⁡_ should be defined to ensure the time efficacy and to control the scale of our ensemble model. At the *t*th time stamp, a new CT should be built with the training set based on Step 1. Meanwhile, the “old enough” CTs should not be discarded.
- Adaptively select the CTs. An accuracy weight of each CT is computed. We then select the CTs based on an adaptive selection method to achieve the optimal CF.
- Constitute the misclassified subset *E* and rare-class subset *R*. According to accuracy weight, we select the misclassified instances and rare-class instances from the old chunks, whose distributions are similar to the testing chunk.
- Classify incoming instances by each CT.
- Vote and obtain the ensemble prediction label. For each testing instance *x*, we obtain the ensemble prediction result by using a voting method, which is based on the accuracy weight.

Our proposed CFIM contains the following key methods.

- Sample method.
- We should collect the misclassified instances and rare-class instances from the “old” chunks which have the similar distribution as the current chunk. According to accuracy weight, we choose the “old” but “useful” chunks to help the training. Misclassified instances are used to reinforce learning of the submodel and construct the strong classifiers. Rare-class instances are used to balance the size of majority class and the rare class. If the size of the rare-class set is too small, we should resample the instants in current chunk to reduce the scale of majority class. *P*_*θ*_^*R*^ is defined as the threshold to ensure the rare-class instances are the proportion of the current chunk. It is especially interesting in our sample method that we choose the chunks with the same distribution as the current chunk to form the above two sets according to accuracy weight in the concept drift environment. Therefore, this method makes full use of the “old” chunks but discards the “useless” chunks.
- Adaptive selection method.
- A new accuracy weight for selection of CTs has been defined to dynamically select “good enough” experts (i.e., CTs) into the optimal CF. Unlike most existing models (e.g., AWE and AUE) where the number of submodels is fixed, the number of CTs in our strategy is varied according to the changes of concepts (see Section 5.2).
- Voting method.
- According to the accuracy weight, we utilize the historical information of the training data to ensure the ensemble prediction capabilities of the submodels.

## 4. Sampling Method

**Figure 2:**
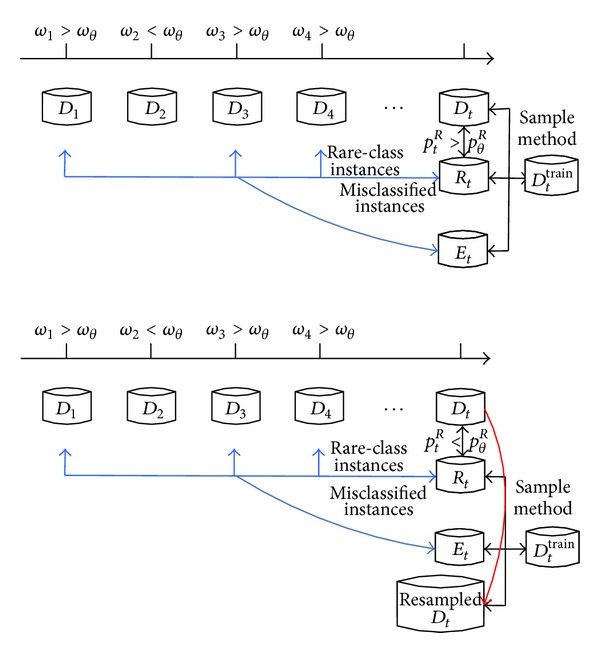
Process of forming the training set.

In this section, we propose the sample method of our CFIM. According to sample method, we combine the instances from misclassified instances *E*_*t*_, rare-class instances *R*_*t*_, and the current chunk *D*_*t*_ into the training chunk *D*_*t*_^train^ at each time stamp. The key procedures of sampling method are to build the misclassified set and rare-class set. The detailed information is shown in Algorithm 2 and Figure 2.

### 4.1. Misclassified Set

To obtain the higher accuracy from the CFIM, we should incrementally emphasize distinguishable ability of the submodels. The instances that previous models misclassified contain more information to enhance the classification accuracy than the accurately classified instances. Therefore, we collect all the instances which are given the false labels into the misclassified set *E*_*t*_ and add *E*_*t*_ into the current training chunk *D*_*t*_^train^. It is worth pointing out that according to accuracy weight in CFIM, all the misclassified instances in *E*_*t*_ are considered to have the similar distribution as the current chunk. If a historical submodel obtains higher accuracy weight than the threshold of accuracy weight (*ω*_*i*_ > *ω*_*θ*_), its corresponding chunk *D*_*i*_ is considered to have the similar distribution as the current chunk. So we accumulate the instances that are misclassified by *f* ^*E*^ in data chunk *D*_*i*_. According to *E*_*t*_, most informative data should be gathered to build the new submodel. Meanwhile, the data related to “old” concept should be discarded.

### 4.2. Rare-Class Set

The number of rare-class instances in the most recent chunk is far from sufficient to train a model with high accuracy in the imbalanced stream environment. The traditional model, ignoring the imbalanced problem, would perform poorly on the rare class. All rare-class instances in the previous chunks associating with the current concept should be accumulated into the training set. In particular, if the proportion of the rare-class instances in the training is too small to build the high-accuracy submodel (*p*_*t*_^*R*^ < *p*_*θ*_^*R*^), we should undersample the majority class instances from the current chunk *D*_*t*_ to balance the class distribution. Figures 2(a) and 2(b) show the process of forming the training set at the recent time stamp under two situations (*p*_*t*_^*R*^ > *p*_*θ*_^*R*^ and *p*_*t*_^*R*^ < *p*_*θ*_^*R*^), respectively.

## 5. Adaptive Selection Method and Voting Method

In this section, we outline another two important methods of our CFIM framework: an adaptive selection method and voting method.

### 5.1. Adaptive Selection Method

Adaptive selection method in our framework seeks to select suitable CTs to organize the optimal CFIM. The CTs related to the current concept are considered as the useful CTs to predict the testing instances. The number of CTs increases when the concept is not changed. However, if the concept drift is detected, most of the old CTs do not represent the new concept, so they are not useful to classify the new testing instances. Only the “useful” CTs can participate in classification.

Algorithm 3 illustrates the adaptive selection method in CFIM. We estimate the accuracy weight *ω*_*i*_ of all the CTs by the new chunk. We then use all the selected CTs (*ω*_*i*_ > *ω*_*θ*_) to classify the testing instance. More remarkably, our method is based on the assumption that the latest CT always participates in prediction.

The following equation is used to compute the accuracy weight:

(1) $$\begin{array}{c} \omega_{i}=\frac{\varphi_{i}}{\sum_{i=1}^{M}\left(\varphi_{i}\right)}, \end{array}$$

where *M* is the total number of CTs, *φ*_*i*_ is the accuracy of each CT. We define the threshold *ω*_*θ*_ which is used to decide whether each CT is suitable or not. The threshold *ω*_*θ*_ is given by

(2) $$\begin{array}{c} \omega_{\theta }=\{\begin{array}{cc} \max\left(\min\varphi_{i}-\tau ,\frac{1}{2}\right) & \bar{\varphi }-\min\varphi_{i}\leq \varepsilon \\ \max\left(\bar{\varphi },\frac{1}{2}\right) & \bar{\varphi }-\min\varphi_{i}>\varepsilon . \end{array} \end{array}$$

Here, the concept drift is detected by the difference between the average accuracy $\bar{\varphi }$ and the minimum accuracy min⁡*φ*_*i*_ ($\bar{\varphi }-\min\varphi$) in the original CF. If concept drift does not occur (i.e., $\bar{\varphi }-\min\varphi_{i}\leq \varepsilon$), each CT takes part in classifying the incoming instances. The parameter *τ* is the minimum value to ensure that all selected CTs can participate in the ensemble model. Otherwise, if the concept drift is detected (i.e., $\bar{\varphi }-\min\varphi_{i}>\varepsilon$), only some “useful” trees with their accuracies higher than $\max(\bar{\varphi },1/2)$ can participate in classification.

### 5.2. Voting Method

We may construct a voting method related to the accuracy weight to integrate results of subclassifiers into an ensemble result. We may use the accuracy weight for each CT as the final voting weight. That is,

(3) $$\begin{array}{c} v_{i}\left(x_{j}\right)=\omega_{i}, \end{array}$$

where *ω*_*i*_ is the accuracy weight of each CT.

The ensemble prediction label *L*_*j*_ of the testing instants **x**_*j*_ can be set to the maximum value of ensemble function *f* ^*E*^(**x**_*j*_).

## 6. Experiments

### 6.1. Datasets

At present, lack of benchmark datasets for textual stream with imbalance class is the problem of classification performance evaluation. In fact, as collecting large-scale real-world textual streams needs to consume huge time and manpower to manually label a mass of instances, it is hard to construct such textual streams. Thus, in order to evaluate the performance of CFIM, we have compiled five imbalanced stream datasets with concept drift. The detailed description is seen in Table 1.

The Spam Assassin Collection (Spam for short) [11] is used as the real-world textual stream. This collection consists of 9,324 instances in Spam class and ham class, and each instance contains 500 attributes. The imbalanced radio of this corpus is 3 : 1. All the instances are arranged in the arrival order. The previous studies [11] show that the attributes of emails gradually change over time.

We construct a new large-scale textual stream (called Spam-Enron stream for short) by the Spam Assassin Collection and Enron Email Dataset with 33,702 instances. This dataset is chronologically collected with 17,157 instances belonging to the Spam class, while other 16,545 ones belonging to the ham class. We use the sigmoid function to formulate a weighted integration of two real-world streams in order to characterize the target concepts in a concept drift environment [7]. Based on this process, we construct the Spam-Enron stream with 100,000 instances, each of which contains 2,044 attributes.

The third stream is collected by the Spam Assassin Collection and a benchmark dataset, Reuters. We sample the instances which belong to the first class and the seventh class in Reuters in order to construct the imbalanced stream (1 : 10). According to the sigmoid function, we create a new stream (called Reuters-Spam stream) with 10,000 instances, each of which contains 19,433 attributes.

Table 1 shows that the Spam Assassin stream and Spam-Enron stream are two imbalanced streams where the proportion of two classes is approximately 3 : 1. We add another imbalanced stream with 1,187 instances in one class and 6,937 instances in the other one to evaluate whether CFIM is suitable for the imbalanced stream. This stream (Spam1 for short) is generated from the Spam Assassin stream by sampling a certain number of instances in the Spam class. The ratio of the number of instances in two classes is approximate to 6 : 1 in the imbalanced Spam Assassin stream.

Similarly, another imbalanced textual stream generated from Spam Assassin stream is collected. This textual stream (Spam2 for short) contains 6,920 instances in one class and 280 instances in another one. The ratio of the number of instances in two classes is approximated to 25 : 1.

### 6.2. Evaluation Procedure

The evaluation procedure is used to arrange the training instances and the testing instances. The basic assumption is that the testing chunk is basically similar to the most recent chunk in the stable period, while the distribution of the testing chunk is changed when a concept drifts. Interleaved chunk method [19] is considered as the evaluation procedure in our paper.

Interleaved chunk combines Holdout and Interleaved Test-Then-Train evaluation methods [19]. The procedure is as follows.

- Collect incoming samples to form a data chunk.
- Test this data chunk to evaluate the latest model.
- Train this data chunk again to update the classification model.

The Spam Assassin stream, Spam1 stream, and Spam2 stream use the interleaved chunk procedure by collecting 300 instances as a textual chunk for one time stamp in a proper sequence. Therefore, Spam Assassin stream, Spam1 stream, and Spam2 stream include 30 time stamps, 26 time stamps, and 23 time stamps, respectively. Similarly, Spam-Enron stream and Reuters-Spam stream, which collect 500 instances as a chunk, include 200 time stamps and 19 time stamps, respectively.

### 6.3. Evaluation Measures

Two important evaluation measures regarding accuracy are used in our paper. To evaluate the overall performance of a learning algorithm, we employ average accuracy. To represent the “accuracy” at each time stamp in an evolving environment, the plotting accuracy is of great importance. The calculations of the average accuracy and the plotting accuracy can be found in [20].

However, the average accuracy and plotting accuracy are not suitable for evaluating the performance of classifiers for an imbalanced data set. The number of instances in the small classes is too small to apparently have effect on the percentage of accuracy. An alternative measure to evaluate the classification error in an imbalanced environment is the kappa statistic [21], which is used to compute the agreement between observed proportion and the expected proportion. The detailed calculation method for kappa statistic is shown in [21].

### 6.4. Compared Models

For comparing the performance, our model has been compared with four state-of-the-art ensemble models, which are much related to our work. These models are Accuracy Weighted Ensemble (AWE) [5], Accuracy Updated Ensemble (AUE) [22], Leveraging Bag (LB) [23], and OzaBagAdwin (OZA) [2]. AWE as a popular method uses the weighted ensemble classifier; AUE extends AWE by using incremental submodels and updating them according to the prediction accuracy; LB combines the Adwin algorithm and the Leveraging Bagging algorithms by using Random Output Codes (ROC). OZA detects and estimates the change by the Adwin algorithm for providing the ensemble weights. In our experiment, we choose Random Tree (RT), Random Forest (RF), libSVM (SVM for short) as basic learners. Since all these three basic learners are used in AUE, AWE, LB, and OZA, respectively, 12 comparative methods take part in the experiments. All these tested algorithms are implemented in the Massive Online Analysis (MOA) platform [7]. We assign the cost function of SVM *c* = 1. The maximum number of subclassifiers in all tested ensemble models to *M*_max⁡_ = 15 in the Spam-Enron stream and to *M*_max⁡_ = 5 in the other streams.

### 6.5. Results

We experiment in 5 textual streams with concept drift under an imbalanced environment. The measures in the comparative experiment involve averaged accuracy, plotting accuracy, and kappa statistic. We outline the experimental results of different approaches regarding the average accuracy for all streams in Table 2. As observed from Table 2, CFIM achieves the highest average accuracy in comparison to all the algorithms in all the streams. Moreover, in five real-world streams under an imbalanced environment, average kappa statistic of the tested approaches in Table 3 shows that CFIM keeps the higher performance in terms of kappa statistic in most streams. We analyze the detailed experimental results in the five textual streams in the following paragraphs.

**Figure 3:**
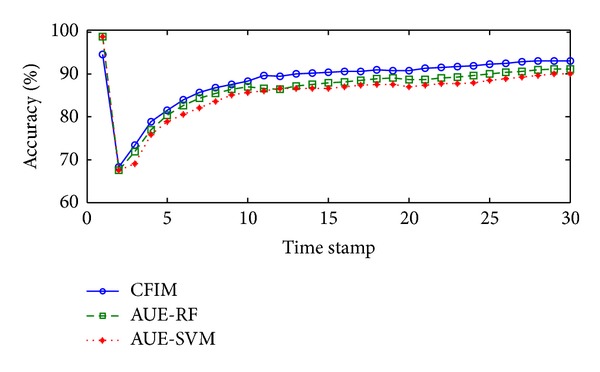
Plotting accuracies in the Spam Assassin stream.

In Spam stream, the average accuracy produced by each algorithm is summarized in Table 2. Compared with AUE-RF, which is the second best algorithm, CFIM achieves 2.09% accuracy improvement. As seen in Figure 3, the curve of plotting accuracy about CFIM is always above the other two curves related to the algorithms with the second and the third highest average accuracy, respectively. At the 3rd time stamp, a large change may have occurred. The curves of all three algorithms tend to gradual increase and are relatively stable after the 3rd time stamp.

**Figure 4:**
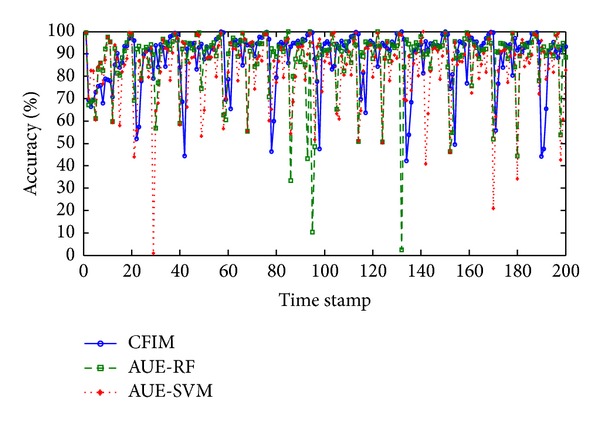
Plotting accuracies in the Spam-Enron stream.

The average accuracy of CFIM in Table 2 is higher than other comparative algorithms in Spam-Enron dataset.  Figure 4 describes the best three algorithms' (CFIM, AUE-RF, and AUE-SVM) plotting accuracies for Spam-Enron dataset. It is worth pointing out that CFIM accomplishes the better performance compared with the other two algorithms, as the fluctuation of accuracy curve in CFIM is the smallest. For example, plotting accuracy curve of AUE-RF fluctuates in a range of (16.6%, 100%) while the curve of CFIM is limited in the range of (49%, 100%). When a concept changes drastically, curves of AUE-SVM and AUE-RF fall down even below the level of CFIM's curve. In the periods of rebuilding concept, all of the three algorithms rebuild the model and tend to be stable. CFIM with a quick substitution of components creates a quick response for new concept, but the plotting accuracy of AUE-SVM grows slowly. This indicates that AUE-SVM is difficult to react to new concepts, especially after a sudden concept drift.

**Figure 5:**
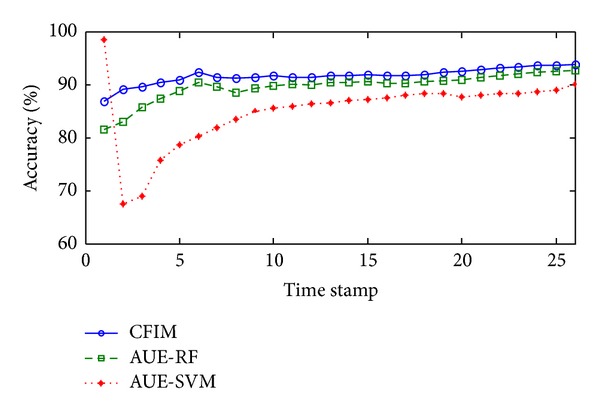
Plotting accuracies in the Spam1 stream.

The average accuracy in the Spam1 stream is shown in Table 2. CFIM gives the best result (91.78%), while AUE-RF (89.75%) is the second best method. The average accuracy of CFIM is 5.84% higher than the third best algorithm, AUE-SVM. The plotting accuracies of three algorithms in the Spam1 stream in Figure 5 show that the curve of CFIM is always above the other two curves. In comparison to the curve of AUE-SVM with the dramatical decrement at the 2nd time stamp, the trends of curves produced by CFIM and AUE-RF seem to be similar. The plotting accuracy is increasing during the first six time stamps. After a slight drop at the 8th time stamp, the curves increase steadily from the 8th time stamp to the 26th time stamp.

**Figure 6:**
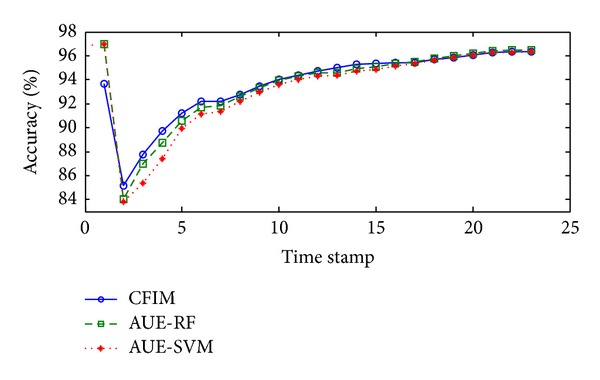
Plotting accuracies in the Spam2 stream.

Likewise, owing to both the highest accuracy and the quick response to sudden drifts, CFIM delivers the best performance in the Spam2 stream as presented in Table 2 and Figure 6. After the accuracies of all the models decrease at the 2th time stamp, all the models tend to ascend their accuracies by rebuilding themselves.

**Figure 7:**
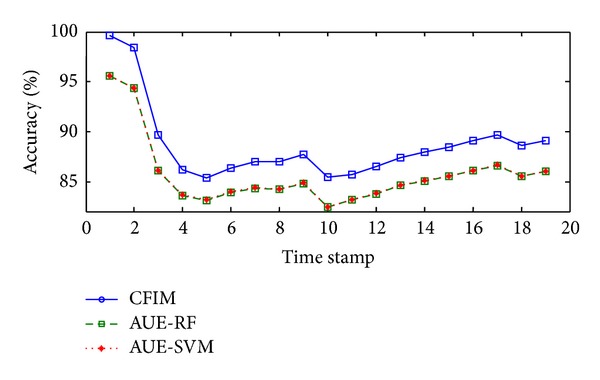
Plotting accuracies in the Reuters-Spam stream.

High-dimensional stream with concept drift is generated from Reuters. As shown in Table 2, CFIM gains the highest average accuracy (88.70%) over all the compared algorithms; AUE-SVM and AUE-RF are the second best algorithms by achieving similar accuracy.  Figure 7 describes the classification results of the Reuters-Spam stream. CFIM shows a strong ability to deal with the concept drift by rebuilding the model quickly with high plotting accuracy.

The kappa statistic results are shown in Table 3. CFIM obtains the highest kappa statistic (70.54%) of all 13 algorithms in the Spam stream. AUE-RF with 65.64% kappa statistic and AUE-SVM with 62.41% kappa statistic are ranked in the second and the third place. Meanwhile, in the Spam1 stream, CFIM, AUE-RF, and LB-RF achieve the highest level of kappa statistic. Though CFIM outperforms the other 12 algorithms with respect to average accuracy, it gains slightly lower (0.6%) kappa statistic than AUE-RF but gains 1.77% higher than LB-RF in the Spam1 stream. Compared with the 12 tested algorithms in the Spam-Enron stream in terms of kappa statistic, CFIM gains the highest performance (62.66%). The similar experimental results are observed in Spam2 stream and Reuters-Spam stream. According to three measures (average accuracy, plotting accuracy, and kappa statistic) to evaluate three imbalanced streams, we can observe that CFIM accomplishes promising performance compared with the other 12 tested algorithms.

## 7. Conclusion

In this paper, we present a new ensemble approach, CFIM, to handle the textual stream classification with class imbalance. This work involves the following aspects.

- We design an adaptive selection method to select the useful CTs and design a voting method depending on the accuracy weight to obtain the ensemble prediction result.
- We construct the training set by sampling the instances from rare-class subset, misclassified subset, and the incoming chunks at a proper radio.
- Experiments on real-world streams are carried out to evaluate the performances of the CFIM, AUE, AWE, LB, and OZA ensemble models based on three evaluation metrics: average accuracy, plotting accuracy, and kappa statistic. The experimental results illustrate that our CFIM approach is more effective than other tested algorithms in most of the streams.

In the future work, we further extend our work in several aspects. First, it is interesting to improve our proposed algorithm by redesigning the structure of CT for multilabel stream classification. Second, we will extend our approach to semisupervised stream classification. Third, we plan to improve our work to design a dynamic streaming clustering tree by an incremental method in order to save the running time.

## Figures and Tables

**Algorithm 1:**
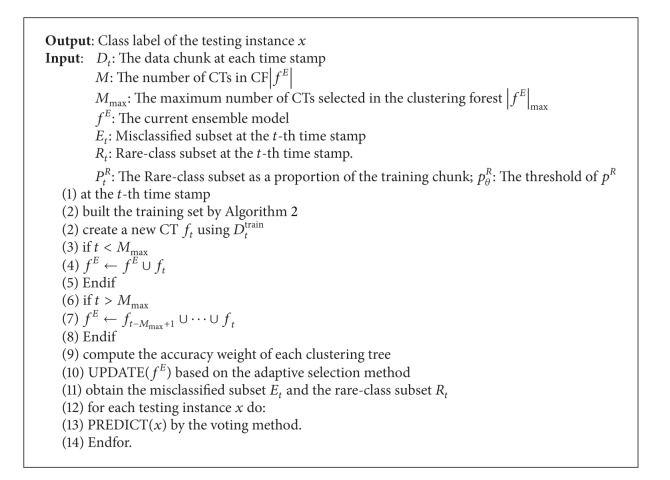
Clustering forest.

**Algorithm 2:**
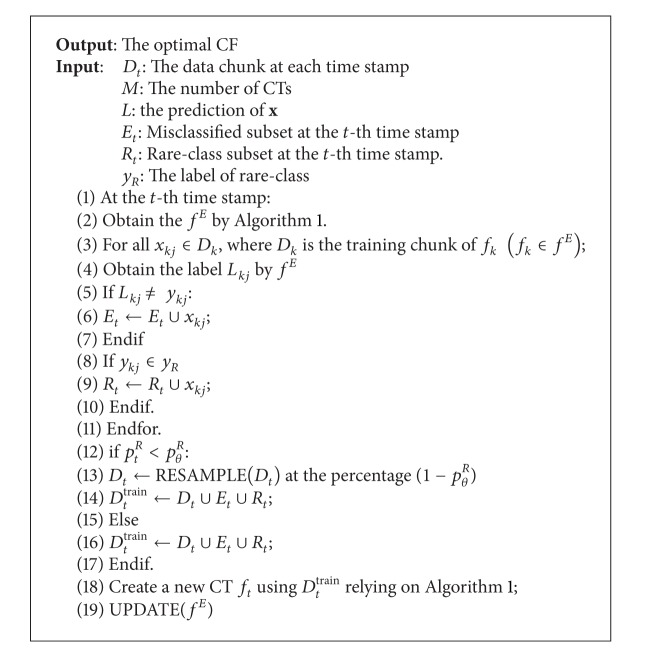
Sample method.

**Algorithm 3:**
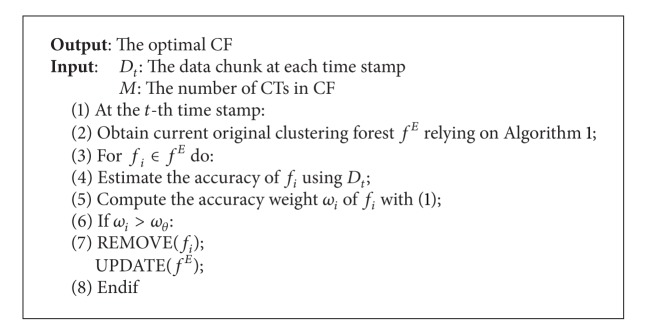
Adaptive selection method.

**Table 1:** The properties of textual streams.

	The number of instances			Imbalanced radio	Size of attribute	Size of each chunk
	Spam	Legitimate	Total			
Spam Assassin	2,387	6,937	9,324	1 : 3	500	300
Enron Email	17,157	16,545	33,702	1 : 1	1,545	—
Spam-Enron	25,000	75,000	100,000	1 : 3	2,044	500
Spam1	1,187	6,937	8,100	1 : 6	500	300
Spam2	280	6,920	7,200	1 : 25	500	300
Reuters-Spam	956	9,044	10,000	1 : 10	19,433	500

**Table 2:** Average accuracy of different algorithms on all streams.

	Spam	Spam-Enron	Spam1	Spam2	Reuters-Spam
CFIM	**88.68**	**88.37**	**91.78**	**93.67**	**88.70**
AUE-RT	**81.73**	**81.37**	**82.24**	88.26	80.40
AUE-RF	**86.59**	**87.28**	**89.75**	**89.68**	**85.72**
AUE-SVM	**85.33**	**85.86**	**85.94**	**89.33**	**85.74**
AWE-RT	77.24	75.71	70.69	46.74	78.31
AWE-RF	**82.45**	78.91	75.54	57.84	84.94
AWE-SVM	**81.11**	72.50	71.38	54.52	**85.27**
LB-RT	64.68	74.65	73.42	87.92	85.26
LB-RF	63.47	79.30	**84.27**	**88.70**	**85.27**
LB-SVM	76.09	72.68	50.69	**88.66**	85.27
OZA-RT	54.34	80.74	75.19	74.59	85.27
OZA-RF	67.80	**81.56**	79.39	74.07	85.27
OZA-SVM	70.88	72.50	78.28	74.33	85.27

**Table 3:** Kappa statistic of different algorithms on all streams.

	Spam	Spam-Enron	Spam1	Spam2	Reuters-Spam
CFIM	**70.54**	**62.66**	**62.31**	**32.81**	**3.89**
AUE-RT	**55.07**	43.67	**50.92**	**31.86**	**3.21**
AUE-RF	**65.64**	**55.96**	**62.91**	**29.85**	**0.38**
AUE-SVM	**62.41**	41.77	**51.37**	**23.12**	0.00
AWE-RT	47.05	42.13	32.43	4.90	**3.24**
AWE-RF	**58.00**	**55.45**	42.36	3.94	**1.88**
AWE-SVM	54.77	35.46	33.21	3.03	0.00
LB-RT	27.50	49.10	39.36	**17.70**	0.01
LB-RF	24.99	**61.44**	**60.54**	0.79	0.00
LB-SVM	45.29	46.78	16.30	0.00	0.00
OZA-RT	14.70	**56.09**	43.89	6.09	0.00
OZA-RF	31.44	**58.76**	54.45	5.92	0.00
OZA-SVM	36.42	53.38	47.32	6.29	0.00
